# Soil Ecological Sustainability in Rice–Crayfish Co-Culture Systems: Bibliometric Patterns and a Narrative Synthesis

**DOI:** 10.3390/biology15212032

**Published:** 2026-11-07

**Authors:** Wei Fu, Changjie Zhang, Qian Yang, Jinchao Han, Bingbing Luo, Ting Yang

**Affiliations:** 1 Ministry of Agriculture and Rural Affairs of the People’s Republic of China Key Laboratory of Sustainable Crop Production in the Middle Reaches of the Yangtze River (Co-Construction by Ministry and Province), College of Agriculture, Yangtze University, Jingzhou 434025, China; weifuu0518@163.com (W.F.); changjie.stu@yangtzeu.edu.cn (C.Z.); 625036@yangtzeu.edu.cn (Q.Y.); 2025710885@yangtzeu.edu.cn (J.H.); 2025720956@yangtzeu.edu.cn (B.L.); 2 Engineering Research Center of Ecology and Agricultural Use of Wetland Ministry of Education, Hubei Key Laboratory of Waterlogging Disaster and Agricultural Use of Wetland, Jingzhou 434025, China

**Keywords:** rice–crayfish co-culture, soil ecology, soil–water–biota nexus, visualization analysis, research trends

## Abstract

Rice–crayfish co-culture (RCC) has become an important integrated rice–aquaculture practice, with potential implications for soil ecological processes and agricultural sustainability. However, the distribution of research attention and the available evidence concerning its soil ecological effects have not been fully integrated. This study combined bibliometric analysis of literature indexed in the Web of Science Core Collection (WoS) and China National Knowledge Infrastructure (CNKI) with a narrative synthesis of soil ecological processes. The datasets comprised 108 WoS records and 273 CNKI records retrieved up to September 2025. Keyword co-occurrence analysis identified 268 nodes and 657 links in the WoS network, compared with 247 nodes and 394 links in the CNKI network. Prominent keywords reflected research attention to soil properties, biodiversity, nutrient cycling, and agricultural production. To organize the ecological evidence, the reviewed literature was discussed across six author-defined themes: soil physical structure and aggregate stability, nutrient cycling and soil fertility, soil organic carbon dynamics, greenhouse-gas emissions, heavy metals and other pollutants, and microbial communities and functions. The narrative synthesis indicates that RCC may influence soil structure, nutrient redistribution, carbon dynamics, redox conditions, and microbial communities through interactions among crayfish activities, organic inputs, and water management. However, the direction and magnitude of these responses depend on environmental conditions and management practices, while potential trade-offs involving greenhouse-gas emissions, contaminant mobility, and soil biodiversity remain insufficiently resolved. Future research should prioritize long-term field observations, integrated assessments of multiple ecological processes, and comparisons across management regimes. By distinguishing bibliometric patterns from ecological evidence, this study provides a structured overview of RCC-related soil research and identifies knowledge gaps relevant to its sustainable management.

## Simple Summary

Rice–crayfish co-culture has expanded rapidly, with reported cultivation exceeding 1.69 million hectares in 2023 and an increasing number of scientific studies. This study analyzed 108 Web of Science records and 273 CNKI records and manually synthesized the literature into six themes: soil structure and aggregation, nutrient cycling and fertility, soil organic carbon, greenhouse-gas emissions, heavy metals and other pollutants, and microbial communities. The findings suggest potential improvements in soil fertility, nutrient availability, and carbon accumulation, alongside possible risks involving soil structure, greenhouse-gas emissions, and pollutant mobility. These benefits and risks depend on environmental conditions and management practices, particularly flooding regimes and organic inputs. Long-term field studies at fixed sites, integrating multiple soil and environmental indicators, are needed to clarify the environmental effects of rice–crayfish co-culture and evaluate its long-term sustainability.

## 1. Introduction

**Figure 1:**
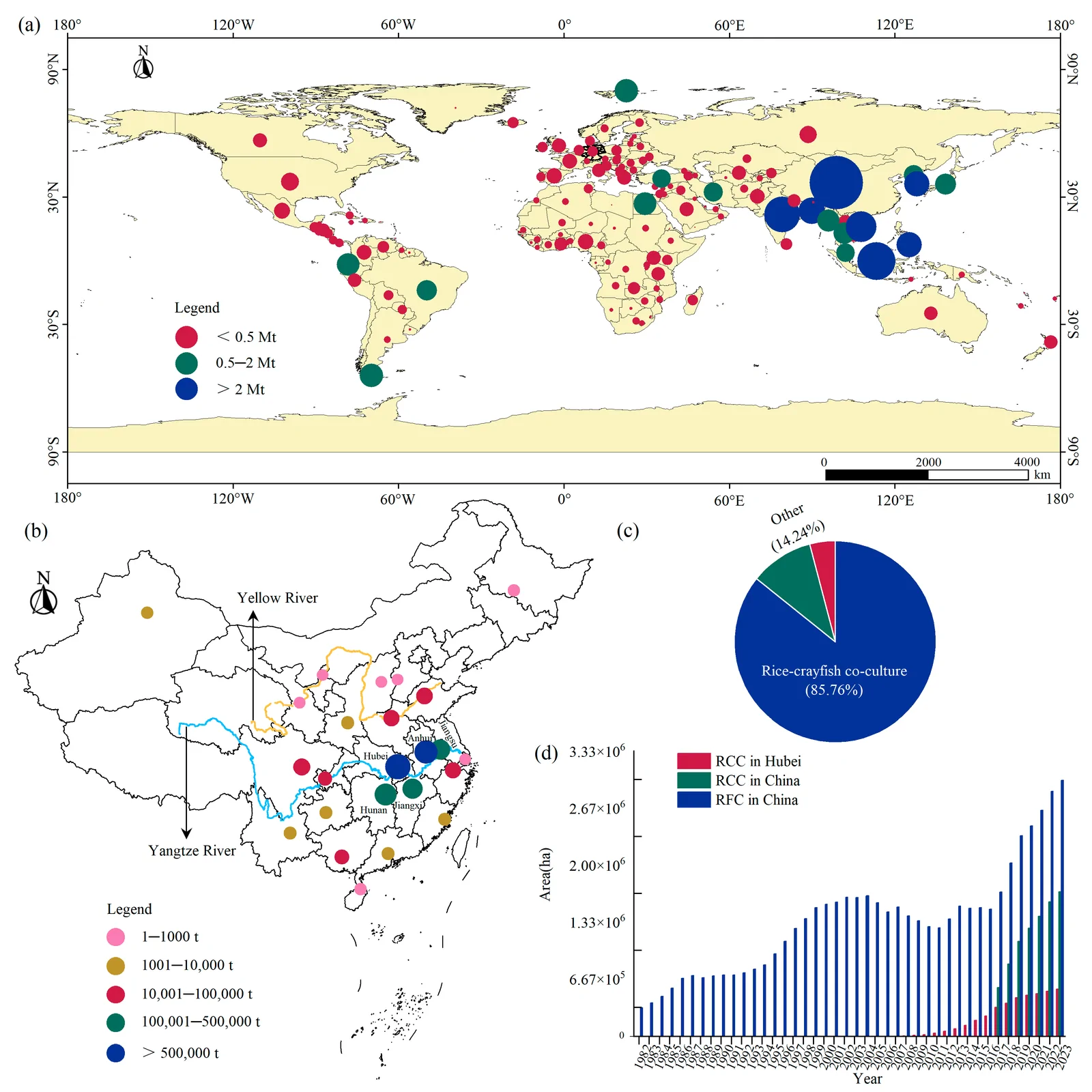
(**a**) Aquaculture production by country in 2023 (bubble size proportional to national aquaculture production; unit: million tonnes, live weight). (**b**) Provincial crayfish aquaculture production in China in 2023 (unit: tonnes). (**c**) Production shares of crayfish farming systems in China in 2023 (rice–crayfish co-culture vs. other systems). (**d**) Changes in the cultivation area of integrated rice–aquaculture farming in China and rice–crayfish co-culture (RCC) in China and Hubei Province from 1982 to 2023. Note: Panel (**a**) is based on data from the Food and Agriculture Organization of the United Nations (FAO), while panels (**b**–**d**) are based on statistics reported in the China Crayfish Industry Development Report (2024). Aquaculture in panel (**a**) refers to farmed aquatic production rather than capture fisheries. Panel (**c**) presents production shares rather than cultivation-area shares. Integrated rice–aquaculture farming in panel (**d**) refers to rice-based farming systems incorporating aquatic animals, rather than rice–fish culture (RFC) alone. Data on the RCC area in Hubei Province from 1982 to 2005 and in China from 1982 to 2016 are unavailable; missing observations should not be interpreted as zero values.

Amid mounting global population pressures and increasing constraints on arable land availability, enhancing productivity per unit area has become imperative for achieving sustainable intensification while safeguarding ecosystem integrity [1,2,3,4]. In parallel with the rapid growth of aquaculture worldwide (Figure 1a), integrated rice (*Oryza sativa* L.)—animal co-culture systems (e.g., rice–crayfish, rice–duck, and rice–fish) have emerged as promising strategies to reconcile agricultural production with environmental sustainability. Beyond generating economic benefits, these systems enhance paddy ecosystem functioning, particularly through improvements in soil health, nutrient cycling efficiency, and physicochemical stability, which underpin long-term environmental performance [5,6,7].

Notably, rice–fish co-culture (RFC) has been practiced for over 2000 years in parts of China (e.g., Sichuan, Guangxi, and Guangdong) [8], whereas the systematic integration of crayfish (*Procambarus clarkii*) into rice-based production represents a more recent agroecological innovation. In China, crayfish aquaculture is spatially concentrated and RCC now contributes the dominant share of national crayfish production (Figure 1b,c), highlighting its rapidly expanding relevance [9]. Owing to their high environmental adaptability, omnivorous feeding behavior, and strong reproductive capacity [7], crayfish have become well established within rice agroecosystems. In RCC systems, crayfish suppress weeds and pests, enhance bioturbation, and contribute organic nutrient inputs via excreta, thereby reducing dependence on synthetic fertilizers and pesticides [10,11]. Meanwhile, paddy fields provide suitable habitats and abundant trophic resources for crayfish growth. This reciprocal interaction forms a circular agroecosystem characterized by dual water use efficiency and dual harvest outputs within a single production unit [12]. Consequently, RCC has evolved into a prominent model of integrated rice–aquaculture systems (Figure 1d) and is increasingly promoted as a pathway toward cleaner production and sustainable agricultural transition.

In recent years, the introduction of crayfish as a managed dominant species into paddy agroecosystems has been shown to markedly alter soil physicochemical properties and ecological functions. Under prolonged flooding and the ridge-ditch configuration typical of RCC systems, the characteristic behaviors of crayfish—feeding, burrowing, and excretion—not only modify soil structure and redox conditions but also reshape the migration and transformation processes of nitrogen, phosphorus, potassium, and organic carbon within the paddy soil environment. Meanwhile, the inputs of feed residues and biogenic detritus alter the nutrient input structure and microbial metabolic pathways, leading to nutrient cycling patterns that differ markedly from those of conventional rice cultivation systems. Although existing studies indicate that RCC has the potential to enhance soil fertility and ecological functioning, substantial uncertainties remain regarding the key processes driving soil changes, the magnitude of their impacts, and their long-term consequences. Recent statistics from the China Crayfish Industry Development Report (2024) indicate that RCC currently represents the dominant integrated rice–aquaculture system in China, with a cultivation area surpassing 25.3 million mu in 2023, or over 56% of the national total. The rapid expansion of this production system highlights the urgent need for systematic evaluation of the ecological effects of RCC. In particular, its long-term impacts on soil quality trajectories, nutrient cycling processes, and microbial community structure have emerged as critical scientific questions in the pursuit of sustainable agricultural development.

In this research field, existing review studies, including traditional narrative reviews and meta-analyses, have systematically examined the agronomic performance, ecological effects, changes in soil physicochemical properties, nutrient cycling processes, and economic benefits of RCC systems [12,13,14]. These studies have laid an important foundation for a comprehensive understanding of the ecological functions and production performance of this integrated farming model. However, existing reviews and meta-analyses have primarily focused on specific agronomic or ecological effects of RCC, while the publication patterns, research collaboration, and keyword structure of the relevant literature have received comparatively limited attention. Bibliometric analysis can complement these studies by quantitatively characterizing the distribution of research activity, identifying frequently studied topics, and providing an overview of the field’s research landscape. Combined with a narrative synthesis of soil ecological processes, this approach offers an additional perspective on existing research priorities and knowledge gaps. Therefore, it is necessary to introduce bibliometric approaches to conduct a quantitative synthesis of research on RCC systems, in order to systematically reveal the knowledge structure, developmental trajectory, and evolutionary characteristics of this research field, and thereby better identify the key scientific questions and future research directions. At present, bibliometric approaches have been widely applied across multiple research fields, including geosciences [15], economics [16], management [17], and ecology [18], and have demonstrated strong applicability and reliability in revealing disciplinary development patterns, identifying research frontiers, and predicting future research trends.

Although previous reviews and meta-analyses have examined the agronomic and environmental implications of RCC, they have primarily focused on specific ecological responses and management practices, with comparatively limited attention to the overall publication patterns, research distribution, and keyword structures of the field. Therefore, a complementary assessment combining bibliometric analysis with a synthesis of soil ecological evidence remains valuable. Accordingly, this study combines bibliometric analysis of WoS- and CNKI-indexed publications with a narrative synthesis of RCC-related soil research. The specific objectives are to: (1) characterize publication patterns, contributing countries and institutions, disciplinary distributions, and keyword co-occurrence networks within the retrieved literature; (2) synthesize reported soil ecological responses across six broad themes encompassing physical, chemical, and biological processes; and (3) identify limitations in the available evidence and propose priorities for future research. By distinguishing bibliometric observations from findings reported in the underlying ecological studies, this work aims to provide a clearer understanding of the research landscape and the potential environmental benefits and trade-offs associated with RCC systems.

## 2. Data Sources and Data Processing

Bibliographic data were retrieved from the Web of Science (WoS) Core Collection and the China National Knowledge Infrastructure (CNKI). The WoS applies strict selection criteria to indexed publications, while CNKI provides extensive coverage of Chinese-language academic literature; therefore, both databases were used to broaden the coverage of the retrieved literature [19]. The literature search was conducted on 10 September 2025. An advanced topic search was conducted in the WoS Core Collection using the query TS = (“rice crayfish” AND “soil”), covering titles, abstracts, author keywords, and Keywords Plus. In parallel, literature retrieval was performed in CNKI using a topic search combining the Chinese terms “稻虾” (rice–crayfish) and “土壤” (soil). No restrictions were imposed on publication year or document type, allowing the inclusion of journal articles, dissertations, and other relevant academic publications. A total of 381 database records were retrieved, comprising 108 records from WoS and 273 from CNKI. The two datasets were analyzed separately using CiteSpace, without systematic deduplication within or between databases. Therefore, the combined count of 381 represents database records. Basic statistical analyses and graphical visualizations were performed using Origin 2024, while CiteSpace (version 6.3.1) was employed to visualize research hotspots. For keyword co-occurrence analysis, the WoS and CNKI datasets were processed separately using CiteSpace, with a time slice of one year and “Keyword” selected as the node type. The project configurations included titles, abstracts, author keywords, and supplementary keywords as term sources. Three node-selection threshold groups were recorded for WoS (2/2/20, 4/3/20, and 4/3/20) and CNKI (2/2/20, 4/3/20, and 3/3/20). The link-control settings included MaxLinksPerNode = 10 and MaxLinksToRetain = 2.5. Pathfinder pruning was enabled for both individual time-slice networks and the merged network. Citation normalization was disabled in the saved configurations, while other network calculations followed the software’s built-in procedures. Betweenness centrality was calculated in CiteSpace based on shortest-path relationships to characterize the bridging roles of keywords within the networks. Origin 2024 was used for descriptive statistics, annual publication trend fitting, and graphical visualization. Keyword frequency filters used for figure presentation were distinguished from the analytical node-selection settings. The WoS and CNKI keyword networks were analyzed separately using the terms recorded in their respective datasets. No additional post-analysis merging of semantically related keywords was performed. Terminological variants were therefore retained as separate entries in the original networks, and the reported frequencies and centrality values reflect these network structures.

The geographical distribution of publications was compiled manually based on the country of the first author’s affiliation. Each record was assigned to one country only, and multinational collaborative publications were counted according to the first author’s country to avoid double counting.

**Figure 2:**
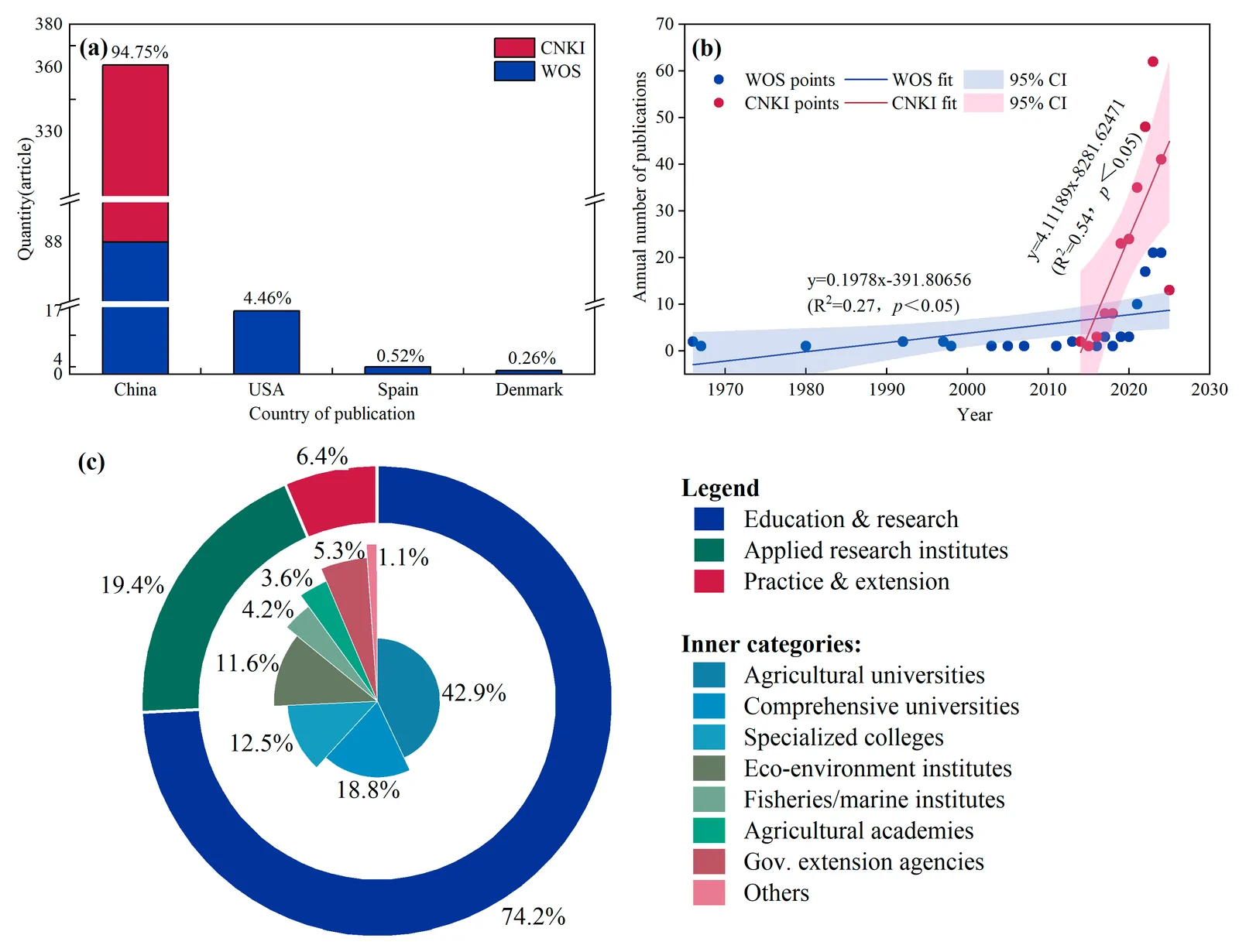
(**a**) Statistical analysis of literature distribution on the effects of Rice–Crayfish Co-culture on soil physicochemical properties. (**b**) Annual Publication Volume of CNKI and WoS. (**c**) Major publishing institutions in China. Note: In panel (**b**), the blue curve represents the linear fit for the WoS dataset, while the red curve represents the linear fit for the CNKI dataset. The publication counts for 2025 are incomplete because the literature search was completed on 10 September 2025. In panel (**c**), the outer ring shows three broad institutional categories, whereas the inner ring presents their subcategories. Government extension agencies (5.3%) are included within the broader practice and extension category (6.4%), rather than representing an additional independent category.

Institutional and disciplinary distributions were calculated using the 381 retrieved database records as the denominator. For institutional classification, each record was assigned to a single category based on the first affiliation of the first author, regardless of additional affiliations or co-authors’ institutions. Thus, multi-institutional publications were counted only once. In Figure 2c, the outer ring represents broad institutional categories, whereas the inner ring shows their corresponding subcategories. Disciplinary classifications were not mutually exclusive, and a single record could be assigned to multiple disciplinary categories.

**Figure 3:**
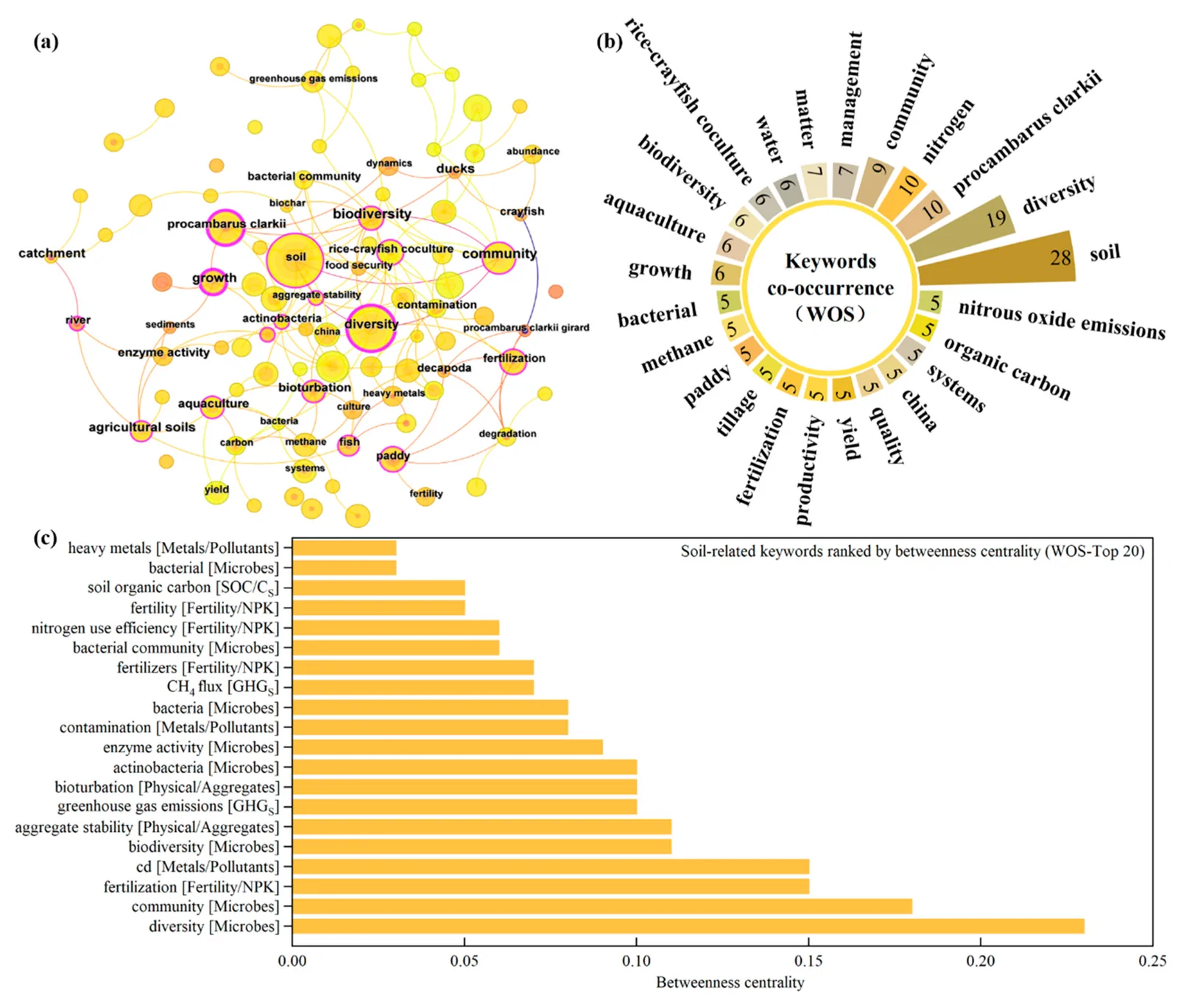
Keyword co-occurrence analysis based on the Web of Science (WoS) dataset. (**a**) Keyword co-occurrence network. Node size represents keyword frequency, and node color indicates the publication period, with cooler and warmer colors corresponding to earlier and more recent years, respectively. (**b**) Radial bar plot of keyword frequencies. (**c**) Representative soil-related keywords extracted from the co-occurrence results, excluding generic terms, and manually organized into six thematic categories: microbial communities and functions, soil organic carbon and carbon stocks, nitrogen–phosphorus–potassium cycling and soil fertility, greenhouse-gas emissions, soil physical structure and aggregate stability, and heavy metals and other pollutants. These categories were defined by the authors to organize the narrative synthesis rather than generated through automated clustering in CiteSpace.

**Figure 4:**
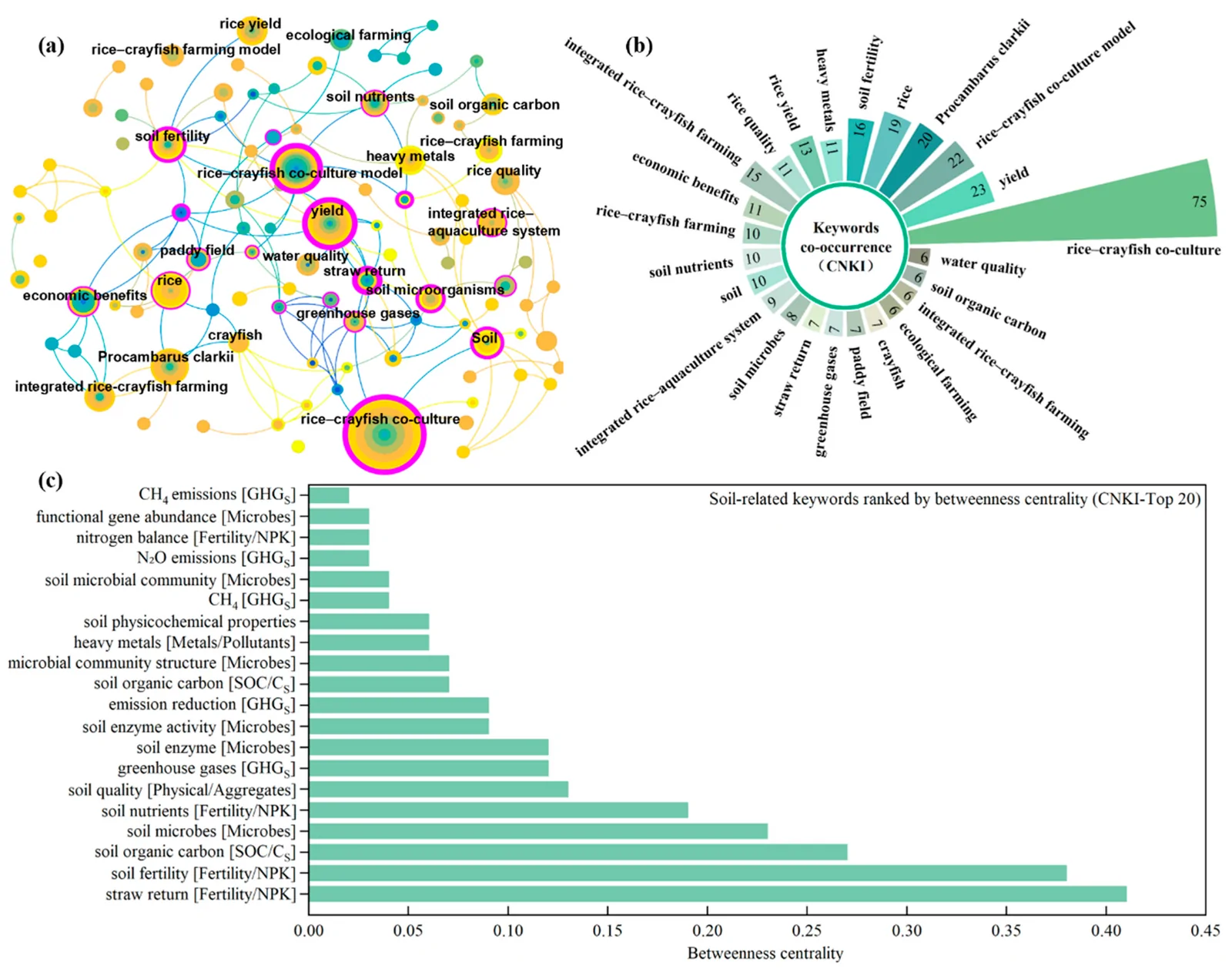
Keyword co-occurrence analysis based on the China National Knowledge Infrastructure (CNKI) dataset. (**a**) Keyword co-occurrence network. Node size represents keyword frequency, and node color indicates the publication period, with cooler and warmer colors corresponding to earlier and more recent years, respectively. (**b**) Radial bar plot of keyword frequencies. (**c**) Representative soil-related keywords extracted from the co-occurrence results, excluding generic terms, and manually organized into six thematic categories: microbial communities and functions, soil organic carbon and carbon stocks, nitrogen–phosphorus–potassium cycling and soil fertility, greenhouse-gas emissions, soil physical structure and aggregate stability, and heavy metals and other pollutants. These categories were defined by the authors to organize the narrative synthesis rather than generated through automated clustering in CiteSpace.

In addition to the bibliometric analysis, a narrative synthesis was conducted to organize the literature on the soil ecological effects of rice–crayfish co-culture (RCC) systems. To provide a clear and systematic discussion, the relevant studies were organized into six broad themes from the perspectives of soil physical, chemical, and biological processes: (1) soil physical structure and aggregate stability; (2) nitrogen, phosphorus, and potassium cycling and soil fertility; (3) soil organic carbon dynamics and carbon stocks; (4) greenhouse-gas emissions; (5) heavy metals and other pollutants; and (6) microbial communities and functions. These themes were defined by the authors based on the principal research topics addressed in the reviewed literature, rather than derived from automated clustering in CiteSpace. Representative keywords were manually assigned to the six themes according to their primary soil ecological meaning and relevance to the reviewed literature. Generic terms with limited thematic specificity were excluded from the thematic presentation. The keyword-to-theme correspondence is illustrated in Figure 3c and Figure 4c. The keyword co-occurrence results were used to provide supplementary context for the thematic synthesis.

The narrative synthesis was conducted separately from the bibliometric analysis and qualitatively organized around the six author-defined soil ecological themes. The synthesis considered the study systems, comparators, geographical locations, soil depths, study durations, management practices, experimental designs, and principal findings reported in the reviewed literature. Direct evidence from rice–crayfish co-culture (RCC) studies was distinguished from contextual evidence derived from other integrated rice–aquaculture systems and general soil-process research. Table 3 summarizes the characteristics and principal findings of four selected field studies supporting the narrative synthesis. Publications outside the bibliometric search cutoff of 10 September 2025, including relevant studies published in 2026, were used as supplementary references for the narrative discussion and were not included in the original WoS or CNKI bibliometric datasets. No quantitative pooling of study outcomes was performed.

## 3. Results and Analysis of Scientific Bibliometrics

### 3.1. Distribution of Research Publications

Based on the reported 381 database records, China accounted for the largest share (361 records, 94.75%), followed by the United States (17 records, 4.46%), Spain (2 records, 0.52%), and Denmark (1 record, 0.26%) (Figure 2a). These figures reflect the geographical distribution of the retrieved records rather than the global distribution of RCC research. Differences in database coverage and indexing practices may influence geographical representativeness. In September 2025, a total of 108 documents were obtained from the WoS database. The annual publication output remained relatively low in the earlier years, followed by an increase in research publications in recent years. The highest annual publication counts were recorded in 2022 and 2023, with 20 articles published in each year. For the China National Knowledge Infrastructure (CNKI) database, a total of 273 documents were collected. The earliest eligible CNKI publication in the dataset appeared in 2015, with an average annual number of publications of approximately 25. The CNKI dataset showed an overall increase in annual publication output during the observed period. Similarly, the WoS dataset exhibited an increase in research publications in recent years (Figure 2b). The fitted curves in Figure 2b were used to describe the overall linear trends in annual publication counts rather than to identify structural changes or distinct developmental stages. Therefore, the fitting results should not be interpreted as evidence of exponential growth or a statistically significant turning point. Furthermore, the publication counts for 2025 are incomplete because the literature search was completed on 10 September 2025. Finally, regarding the distribution of publishing institutions (Figure 2c), higher education institutions accounted for 74.2%, followed by applied research institutions (19.4%) and practice and extension organizations (6.4%). Government extension agencies accounted for 5.3% and were included within the broader practice and extension category rather than treated as an independent category. These results describe the institutional composition of the retrieved literature but do not independently demonstrate policy linkages. In summary, the retrieved literature reflects increasing research attention to RCC-related soil studies, with a strong application-oriented focus and contributions from multiple disciplinary fields.

### 3.2. Journal Analysis

The retrieved literature covered multiple disciplinary fields, including agricultural resource utilization (59.84%), biological sciences (30.18%), environmental science and engineering (27.30%), interdisciplinary studies (13.91%), food science and engineering (9.45%), aquaculture (8.14%), and other topics (6.56%) (Table 1). These percentages were calculated using the 381 retrieved database records as the denominator. Because individual records could be assigned to multiple disciplinary categories, the percentages were not mutually exclusive. Relevant studies appeared in journals such as Soil and Tillage Research, Agriculture, Ecosystems & Environment, Journal of Cleaner Production, Science Bulletin, Journal of the Science of Food and Agriculture, Aquaculture, and Agricultural and Forest Meteorology, as well as in Chinese-language journals and postgraduate theses. These journals are presented as examples of publication outlets rather than ranked by publication counts. Overall, the disciplinary distribution highlights the multidisciplinary nature of RCC-related soil research but does not establish a chronological progression among research fields. Table 2 presents selected publications related to RCC and integrated rice–aquaculture systems, identified with reference to citation rankings in the WoS and CNKI datasets in September 2025. The table is intended to provide an overview of relevant publications rather than establish a formal citation-based ranking or highly cited status. Broader rice–fish reviews and rice–crayfish–turtle studies are included to provide contextual information on integrated rice–aquaculture systems and are not treated as direct evidence of soil ecological responses in RCC systems.

### 3.3. Keyword Co-Occurrence Analysis

Keywords often highlight the core content and central themes of each article. They help readers quickly understand the main research areas, methodologies, key findings, or innovations of the study. Through an analysis of high-frequency keywords in studies on the effects of RCC on soil based on the WoS, the keyword co-occurrence network exhibited a relatively sparse structure (N = 268, E = 657, Density = 0.0184) (Figure 3). To avoid visual clutter, a display threshold was applied in Figure 3, and only keywords with frequencies greater than 1 were retained for visualization. The WoS keyword co-occurrence network contained 268 keyword nodes (Figure 3a). Four keywords occurred at least 10 times. The five most frequent keywords were soil (28 occurrences, betweenness centrality [BC] = 0.17), diversity (19 occurrences, BC = 0.23), *Procambarus clarkii* (10 occurrences, BC = 0.01), nitrogen (10 occurrences, BC = 0.24), and community (9 occurrences, BC = 0.18), respectively. The network node count represents the total number of keyword nodes, whereas the frequencies reported here describe selected high-frequency keywords rather than an exhaustive classification of all nodes.

The construction of the CNKI keyword co-occurrence network (N = 247, E = 394, Density = 0.013) followed the same procedure as that used for the WoS dataset (Figure 4). Among the 247 keywords, 14 had frequencies greater than or equal to 10, 89 keywords occurred between 2 and 9 times, and 144 keywords appeared only once. The top five keywords were rice–crayfish co-culture (75 times, BC = 0.51), yield (23 times, BC = 0.52), rice–crayfish co-culture model (22 times, BC = 0.47), *Procambarus clarkii* (20 times, BC = 0.1), and rice (19 times, BC = 0.11), respectively.

Moreover, we further extracted high-frequency keywords closely related to soil processes from the keyword co-occurrence outputs of the two databases, excluding generic terms with limited thematic specificity (e.g., soil, rice, and system). Keyword frequency and betweenness centrality were used to characterize research attention and the structural positions of keywords within the co-occurrence networks (Figure 3 and Figure 4). To facilitate a systematic discussion of the soil ecological effects of RCC, the relevant literature was organized by the authors into six broad themes based on the principal research content from physical, chemical, and biological perspectives: (1) soil physical structure and aggregate stability; (2) N–P–K transformations and soil fertility dynamics; (3) soil organic carbon processes and changes in carbon stocks; (4) greenhouse-gas fluxes and their drivers; (5) the behavior and ecological risks of heavy metals and other pollutants; and (6) microbial community structure and functional responses. These themes represent an author-defined framework for organizing the narrative synthesis rather than clusters automatically generated by CiteSpace. Notably, prominent keywords in the CNKI dataset primarily reflect system descriptions and agronomic performance, whereas those in the WoS dataset include terms related to nutrient cycling, biodiversity, and microbial communities. Although the keyword co-occurrence networks provide insights into the distribution of research attention, their structural characteristics do not directly establish ecological interactions or causal mechanisms. Moreover, the relatively sparse connections between keyword groups may partly reflect differences in terminology and network filtering settings, rather than necessarily indicating weak relationships among the underlying soil ecological processes. Therefore, further experimental evidence and integrated investigations are needed to clarify the underlying processes and mechanisms through which RCC influences soil ecological functioning.

## 4. Current Perspectives and Challenges

**Figure 5:**
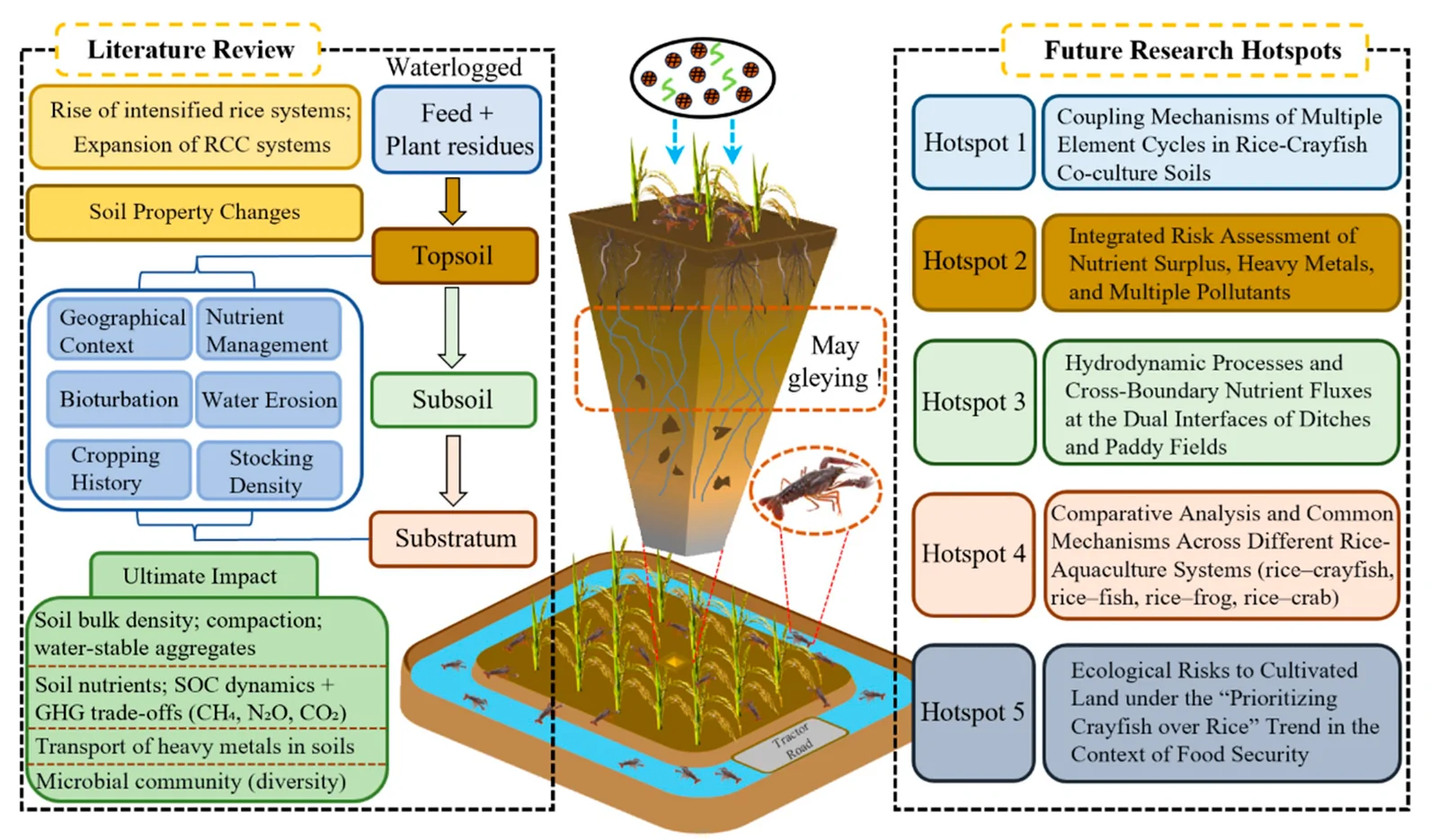
Conceptual synthesis of soil ecological effects and future research priorities in rice–crayfish co-culture (RCC) systems. The left panel summarizes management factors and soil ecological responses discussed in the literature, while the central illustration represents potential interactions across soil layers. Reported findings are synthesized in Section 4, with examples of supporting field studies summarized in Table 3. Proposed mechanistic pathways are interpreted as hypotheses requiring further investigation rather than established causal relationships. The right panel presents future research priorities identified through the narrative synthesis, rather than experimentally validated outcomes or direct outputs of CiteSpace clustering.

Based on the preceding analysis, this chapter focuses on the multidimensional impacts of RCC on the soil environment and is organized around the thematic structure revealed in Figure 3 and Figure 4, proceeding to systematic reviews of the six sub-themes outlined above. To bridge these themes with a process-oriented framework spanning management drivers, soil-layer responses (topsoil–subsoil), and ultimate ecological outcomes, and to highlight key future research hotspots, an integrative conceptual schematic is provided in Figure 5. Within each section, we will synthesize the major findings and their supporting evidence, compare inconsistencies and points of contention across studies, and—within the limits of the available evidence—distill the key drivers and plausible process mechanisms, with the aim of developing a clearer and testable mechanistic understanding of the soil ecological effects of the RCC. Notably, RCC is not simply a fertilization or soil-loosening practice; by introducing a new biotic component (crayfish) into paddy fields, together with its associated management, the system can induce integrated changes in soil physical structure, elemental cycling, and biological communities, thereby shaping soil quality trajectories and associated ecological functions. Under appropriate management, these changes have the potential to enhance soil vitality and system resilience while aligning production objectives with environmental goals.

**Table 3:** Verified study characteristics and main findings in rice–crayfish soil research.

Study	Study System	Comparator	Location	Soil Depth	Study Duration	Management	Study Design	Principal Findings	Evidence Role
Zhang et al. 2025 [32]	Rice–crayfish co-culture (RCC)	RCC fields with 4 versus 8 years of use	Yunlianghu Management District, Qianjiang, Hubei, China	0–10, 10–20, 20–30, 30–40, 40–80, 80–120 cm	RCC history: 4 versus 8 years; sampled May 2024	Rice–crayfish farming; contrasting years of continuous use	Comparative field-profile sampling; soil aggregates and SOC fractions measured	Longer RCC duration was associated with depth-dependent changes in water-stable aggregates and carbon fractions.	Direct RCC; profile comparison
Liu et al. 2022 [33]	RCC with peripheral trench	Rice monoculture	Yimin Town, Houhu District, Qianjiang, Hubei, China	0–20 cm topsoil characterized; NH_3_ flux measured in field and trench	2018 and 2019 rice-growing seasons; RCC site in use since 2010	Fertilized rice cultivation; peripheral drainage/culture trench in RCC	Two-season comparative field experiment; NH_3_ flux, urease activity and rice N uptake	Compared with monoculture, RCC reduced total NH_3_ volatilization by 16.1–19.6% and increased rice N uptake by 16.7–18.3%.	Direct RCC; field comparison
Study on nutrient accumulation, 2021 [34]	Rice–crayfish rotation (source terminology)	Rice monoculture (32 fields) versus rice–crayfish (69 fields)	Jianghan Plain, China	0–20 cm	Farming histories spanning 0–23 years	Rice cultivation; crayfish feed, fertilizer and straw management	Field survey of 101 fields; nutrient input–output budgets and soil sampling	Nutrient surpluses accumulated in the rice–crayfish system; soil total N and readily oxidizable C increased in 0–20 cm soil.	Direct RCC-related; field survey
Zhang et al. 2022 [11]	Rice–crayfish farming	Rice monoculture versus RCC durations	Nanxian County, Hunan, China	0–30 cm	RCC history: 1, 6, 10 and 15 years	Long-term rice–crayfish farming and associated paddy flooding	Space-for-time comparison; soil SOC, water-stable aggregates and reducing substances	Six–15 years of RCC were associated with higher SOC and aggregate stability, together with stronger gleying indicators.	Direct RCC; temporal comparison

Note: The four studies are cited as References [32], [33], [34], and [11], respectively, in the main manuscript. The table summarizes their study systems, comparators, locations, soil depths, durations, management practices, study designs, principal findings, and evidence roles. RCC denotes rice–crayfish co-culture.

### 4.1. Soil Physical Structure and Aggregate Stability

#### 4.1.1. Soil Bulk Density and Pore Characteristics

Soil bulk density (BD) is widely used as an indicator of soil quality and serves as an indirect measure of soil aeration and water-holding capacity [35]. Previous studies have shown that, under comparable parent material and tillage regimes, intensive burrowing and foraging activities of crayfish substantially modify surface soil structure. As a result, soil porosity in the 0–10 cm layer is significantly increased, accompanied by the most pronounced reduction in BD. In the 10–20 cm plough layer, BD shows a moderate decrease due to the combined effects of biological penetration and hydrodynamic processes. In contrast, below 20 cm depth, where crayfish activity is limited, BD generally remains comparable to that of conventional paddy soils [36,37]. In addition, the effects of RCC on soil bulk density (BD) may vary with cultivation duration and management practices. Long-term rice–crayfish farming can alter soil carbon storage and related soil properties, while the responses of soil structure depend on site-specific conditions and management regimes [36]. Crayfish burrowing, prolonged flooding, and organic matter inputs may influence soil pore formation and redistribution, although their relative contributions to BD changes require further investigation. Previous research has also demonstrated that watercourse design can influence crayfish burrowing activity and dispersal behavior [38]. Moreover, stocking practices, feed inputs, and field water management may affect soil physical conditions, but the relationships between stocking intensity, soil depth, and long-term BD responses have not yet been sufficiently established. Therefore, further field studies are needed to quantify these relationships under different soil and management conditions. Tillage practices and crop rotation regimes are also non-negligible factors. Variations in ploughing depth, the implementation of field drying, and rice–crayfish-upland crop rotation patterns can all influence soil compaction and loosening processes. Consequently, changes in soil BD under RCC systems arise from the combined effects of biological disturbance, hydrodynamic processes, and field management practices. At present, most studies on soil BD under RCC systems focus on short-term periods of 1–4 years, while continuous monitoring data exceeding eight years remain extremely limited. Consequently, the long-term ecological feedbacks of this system are still poorly understood. In addition, substantial differences in background soil conditions among study regions—such as soil texture (e.g., clay loam versus sandy loam), parent material, and baseline BD—reduce the comparability of experimental results across sites. This heterogeneity limits a deeper understanding of the mechanisms and long-term trajectories through which RCC influences soil properties.

#### 4.1.2. Soil Compaction Dynamics

Soil compaction is widely regarded as a key indicator of soil degradation [39]. In RCC systems, water management differs between rice-growing platforms and crayfish refuge trenches and varies across cultivation stages. Relatively deeper water in refuge trenches provides an aquatic habitat for crayfish, whereas water depth on rice-growing platforms is managed according to crop growth requirements [40]. Therefore, a single water-depth range should not be applied uniformly to the entire paddy field. Existing studies suggest that RCC may influence soil physical conditions through changes in biological activity, water management, and field operations, although the resulting soil responses vary among management systems. Some studies have suggested that changes in machinery use and field operations may affect mechanical disturbance and soil compaction under RCC. Meanwhile, prolonged flooding and puddling may alter soil particle arrangement, pore continuity, and structural stability. Studies of mechanically disturbed soils have demonstrated changes in soil structure under external loading [41], while investigations of paddy soils have shown that puddling intensity influences soil structural properties and crack development [42]. These findings provide a general mechanistic basis for understanding possible structural changes in RCC soils, although they do not directly demonstrate progressive subsoil densification under RCC management. Conversely, soil water conditions can substantially influence soil mechanical responses. Changes in soil moisture and interparticle interactions may affect soil plasticity, penetration resistance, and susceptibility to compression [43,44]. However, lower penetration resistance or greater deformability under wet conditions does not necessarily indicate reduced soil compaction. Actual soil densification should be evaluated using structural indicators such as bulk density and pore characteristics, together with measurements of soil water content. Therefore, findings from unsaturated soil mechanics should be interpreted cautiously when applied to flooded paddy soils. Meanwhile, crayfish burrowing activities and rice-root development may contribute to changes in near-surface soil structure and associated biological processes [45,46]. However, the extent to which these processes directly reduce soil bulk density or persistent compaction under RCC requires further experimental verification. Overall, RCC-related changes in soil physical conditions may result from interactions among biological disturbance, water management, soil mechanical behavior, and cultivation practices. Although existing research provides evidence of changes in soil properties within cultivated soil layers, direct measurements of compaction processes in deeper soil layers (>40 cm) remain limited. Further studies integrating bulk density, penetration resistance, soil moisture, and pore characteristics are therefore needed to clarify the mechanisms of soil structural change and their implications for water–air transport and crop root penetration.

#### 4.1.3. Soil Water-Stable Aggregates: Size Distribution and Stability

Water-stable soil aggregates play a critical role in maintaining soil fertility, enhancing resistance to erosion, and regulating soil aeration and water infiltration [47]. Previous studies have reported that rice–crayfish co-culture (RCC) can increase the proportion of water-stable macroaggregates (>250 μm), indicating changes in soil aggregate-size distribution and potentially improved aggregate stability under certain management conditions [32,48]. However, an increase in macroaggregate abundance does not necessarily indicate structural imbalance or greater aggregate fragility. Aggregate formation and turnover involve interactions between macroaggregates and microaggregates, which are influenced by organic matter, soil mineral composition, and environmental conditions [49]. Under RCC management, prolonged flooding, periodic hydrodynamic disturbance, and biological activity may influence aggregate formation, breakdown, and reassembly [50]. Changes in aggregate-size distribution may also affect soil pore architecture, although the consequences for pore connectivity and hydraulic conductivity depend on the spatial arrangement and continuity of pores. Studies of soil structural processes have demonstrated that variations in aggregate organization and pore characteristics can influence water movement and drainage [51,52]. Nevertheless, changes in macroaggregate abundance alone cannot establish corresponding increases or decreases in hydraulic conductivity, and direct measurements are required to evaluate these relationships in RCC soils. Wet–dry cycles represent another important factor influencing soil aggregate stability and physical properties. During drying, soil water loss may induce capillary stresses, aggregate shrinkage, and internal structural deformation [53,54]. Subsequent rewetting can generate swelling and repeated shrinkage–swelling stresses, potentially affecting aggregate integrity and structural rearrangement [55,56]. These processes provide a general mechanistic basis for understanding soil structural responses to fluctuating moisture conditions, although their magnitude and direction under RCC depend on soil properties and water-management regimes. In addition to physical restructuring, alternating oxic and anoxic conditions can influence iron and manganese transformations in paddy soils. Previous studies have demonstrated that redox fluctuations affect the transformation and redistribution of iron minerals, which are closely associated with soil redox characteristics [57]. Importantly, direct evidence from RCC systems has shown that long-term rice–crayfish farming can aggravate soil gleying and alter soil iron morphology [58]. This finding supports the occurrence of gleying-related changes under prolonged RCC management but does not establish aggregate redistribution or altered pore connectivity as their sole causal drivers. Furthermore, studies of RCC soil profiles have documented variations in soil aggregate distribution and organic carbon with depth [21]. General soil-structure research has also shown that moisture fluctuations and associated physical stresses can contribute to crack development and structural reorganization [59]. Together, these findings suggest that soil aggregation, pore characteristics, water movement, and redox processes may interact under RCC management. However, the relationships among these processes should be distinguished from directly measured outcomes, particularly where pore connectivity and hydraulic conductivity have not been simultaneously evaluated. Future research should therefore focus on identifying the mechanisms linking aggregate dynamics with soil hydrological and redox processes, examining responses across deeper soil layers, and determining how cultivation duration, flooding regimes, and field management influence the development and extent of gleying.

### 4.2. N-P-K Transformations and Soil Fertility Dynamics

Rice–crayfish co-culture (RCC) influences soil fertility by modifying nutrient inputs, water management, and nutrient cycling processes. Although RCC management may reduce mineral fertilizer application under certain conditions, this does not necessarily indicate a reduction in total nutrient inputs, because crayfish feed, feed residues, excreta, and crop residues also contribute nutrients to the system [60,61]. These nutrient sources are spatially and temporally heterogeneous, which may create differences between nutrient availability and rice requirements for nitrogen (N), phosphorus (P), and potassium (K) across growth stages. Consequently, appropriate mineral fertilizer management remains important for maintaining rice production and limiting nutrient surpluses [62]. Understanding the balance between mineral fertilizer inputs, feed-derived nutrients, and nutrient losses is therefore essential for evaluating soil fertility under RCC.

Crayfish feed and associated organic inputs may contribute to changes in soil N availability and accumulation [63,64]. However, increased soil N content alone cannot demonstrate enhanced biological N fixation or a specific symbiotic association between crayfish and N-fixing bacteria. Establishing such mechanisms requires direct evidence of microbial N-fixation activity and its contribution to soil N inputs. Previous RCC field studies have reported reduced ammonia volatilization and increased rice N uptake under particular experimental conditions [33,65]. These measured responses should be distinguished from potential explanations involving field water management and the trench–ridge configuration. Differences in water depth, exposed water surface, N concentration, and hydrological exchange between trenches and rice-growing areas may influence ammonia volatilization and N redistribution, but their respective contributions require direct assessment. In addition, ammonium nitrogen (NH_4_^+^–N) accumulated in refuge trenches may be redistributed through water exchange or lateral transport; however, the extent to which this process supplies N to rice during later growth stages depends on hydrological connectivity, N transformation, and crop demand [33,66]. Therefore, the proposed nutrient-buffering function of trenches should be regarded as a potential mechanism rather than an assured source of late-season N supply.

RCC influences soil P and K cycling through nutrient inputs, water management, and biological processes [25,67]. Feed is an important external P source in fed RCC systems, but its contribution depends on treatment, cultivation period, and nutrient-budget boundaries; therefore, the previously reported contribution exceeding 90% cannot be generalized to all RCC systems [37,68]. In contrast, crayfish excreta, molts, and decomposing residues contribute primarily to internal nutrient recycling. Organic P mineralization and reductive dissolution of Fe oxides may promote short-term P release [69]. However, long-term RCC can reduce available P despite total P accumulation, indicating that P release must be considered alongside adsorption, precipitation, and immobilization [40]. Unlike organic P mineralization, K availability is regulated by residue and mineral K release, exchange, desorption, fixation, and weathering. Crayfish activity and changes in soil structure may further influence nutrient redistribution, although these mechanisms require direct evaluation [70].

In summary, the RCC system drives N, P, and K migration, transformation, and redistribution across various forms and spatial scales by systematically restructuring nutrient inputs, hydrological regimes, and bioturbation processes in paddy fields, thereby fundamentally shaping soil fertility dynamics and crop nutrient supply pathways. It is noteworthy that feed-dominated external inputs often lead to pronounced N, P, and K surpluses in paddy fields. Without effective regulation, such long-term accumulation of nutrient surpluses may not only increase the risk of water eutrophication but also disrupt soil nutrient balance, posing potential challenges to system sustainability [34,71].

### 4.3. Soil Organic Carbon Turnover and Stocks

Soil organic carbon (SOC) depletion in agricultural soils is an important concern for soil fertility and climate regulation [72,73]. Under rice–crayfish co-culture (RCC), carbon inputs from feed residues, crayfish excreta, molts, and plant residues may influence soil carbon cycling [55]. Feed represents an external carbon input, whereas subsequent redistribution through crayfish metabolism and residue decomposition constitutes internal recycling and should not be counted as additional external inputs [11,74]. These organic inputs may also promote aggregate formation and physical protection of SOC [20,75,76].

Importantly, SOC concentration, SOC stock, stock-change rate, and persistent carbon sequestration represent distinct measures. SOC stocks depend on soil depth, bulk density, and the soil-mass basis used for comparison; therefore, increases in SOC concentration alone cannot establish greater carbon stocks or long-term sequestration. Microbial decomposition and dissolved organic carbon (DOC) production contribute to carbon transformation [77,78]. Although changes in DOC, microbial biomass, and enzyme activity may indicate altered microbial processes [79,80], they do not independently demonstrate persistent SOC stabilization. Direct RCC evidence has linked increased SOC stocks with aggregate protection and microbial necromass accumulation [20]. Nevertheless, the long-term persistence of these carbon pools and their association with soil minerals require evaluation using comparable soil depths, preferably equivalent soil-mass approaches, and repeated measurements over time.

### 4.4. Greenhouse-Gas Fluxes and Their Drivers

Rice–crayfish co-culture (RCC) may enhance soil organic carbon (SOC) storage under certain management conditions, but increased SOC stocks do not necessarily indicate net climate mitigation [81,82]. RCC management can alter fertilizer use, ammonia (NH_3_) volatilization, and greenhouse-gas emissions [83]. Although NH_3_ is not a direct greenhouse gas, its emissions may contribute indirectly to nitrogen-related environmental impacts. Potential life-cycle benefits from reduced fertilizer use should also account for upstream emissions associated with fertilizer production, feed inputs, and other management activities [84].

Importantly, prolonged flooding may increase methane (CH_4_) emissions, potentially offsetting reductions in nitrous oxide (N_2_O). Ma et al. [85] reported increased global warming potential (GWP) under RCC because greater CH_4_ emissions outweighed lower N_2_O emissions. Other studies have also highlighted the importance of water management and cultivation practices in regulating CH_4_ emissions [86,87]. Therefore, comparisons of climate impacts should use consistent reference systems, accounting periods, system boundaries, and functional units. Overall, the climate-mitigation potential of RCC depends on management practices and environmental conditions and should be assessed by integrating SOC stock changes, greenhouse-gas emissions, and relevant upstream inputs rather than assuming universal low-carbon benefits.

### 4.5. Heavy Metal Migration

Heavy metal(loid) accumulation in agricultural soils may pose ecological and food-safety risks [88]. In RCC systems, the mobility and accumulation of Cd, Pb, As, and other elements depend on their chemical properties, soil conditions, feed inputs, and water management. Evidence from rice–fish systems provides useful context but should be distinguished from direct RCC findings [89,90]. Feed, water inputs, and crayfish-related nutrient recycling may influence element redistribution, although responses differ among elements and farming systems [91,92].

Flooding and redox fluctuations may alter metal(loid) speciation, while dissolved organic matter can either immobilize or mobilize certain elements depending on environmental conditions [93,94,95]. Importantly, soil metal extractability, concentrations in edible rice grains or crayfish tissues, and dietary health risks represent different endpoints. RCC studies have assessed metal(loid) accumulation and potential dietary risks under specific conditions [93,94,96], whereas rice–fish observations provide contextual evidence [97]. Compliance with food-safety limits must be evaluated separately for each element and edible tissue, considering the applicable standard, chemical species where relevant, and fresh- or dry-weight reporting basis. Therefore, the available evidence does not support a general conclusion that RCC products consistently remain below regulatory limits. Further research should evaluate element-specific transport, bioaccumulation, and dietary exposure across different sites, management practices, and cultivation durations.

### 4.6. Changes in Microbial Diversity

Soil microbial responses to rice–crayfish co-culture (RCC) vary with microbial groups, diversity metrics, farming practices, and cultivation duration. Previous studies have reported changes in bacterial community composition and diversity under RCC management [98,99,100]. However, these responses are not uniformly negative. For example, rice–crayfish rotation has been associated with higher ACE and Chao1 richness indices without significant changes in Shannon or Simpson diversity indices. Such differences indicate that microbial richness and diversity or evenness should be interpreted separately, considering the comparator and management duration.

**Figure 6:**
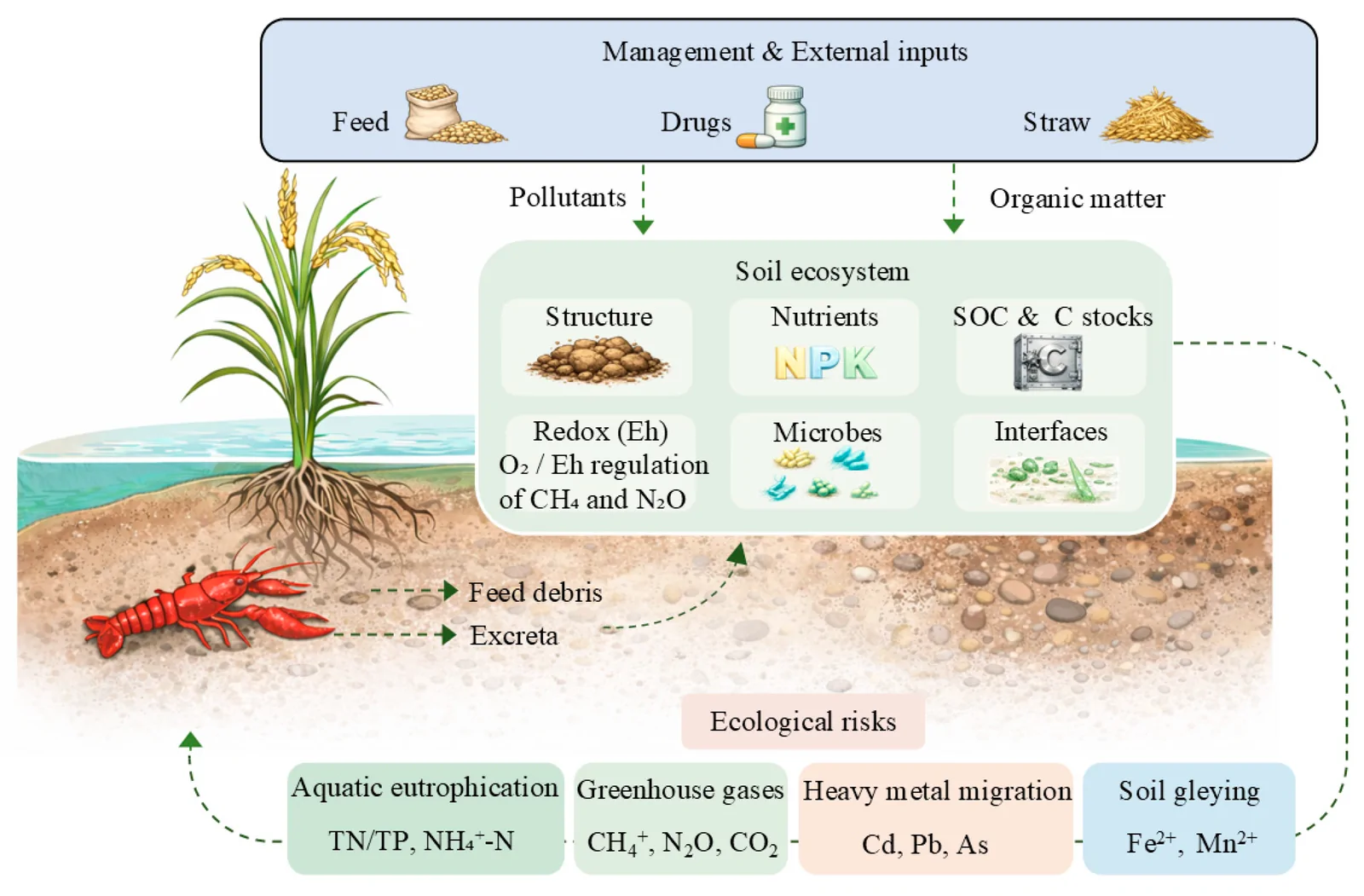
Conceptual framework linking management inputs, soil ecological processes, and potential environmental risks in rice–crayfish co-culture (RCC) systems. Management inputs and internal nutrient transfers are distinguished from soil responses and ecological effects. Redox conditions regulate CH_4_ and N_2_O production and consumption rather than representing direct chemical conversion from O_2_. Dashed arrows indicate potential indirect or hypothesized pathways, rather than experimentally established causal relationships. Whereas Figure 5 summarizes soil ecological responses and future research priorities, Figure 6 focuses on the potential linkages between management practices, soil processes, and environmental risks.

Crayfish activity, prolonged flooding, nutrient inputs, and changes in soil physicochemical conditions may influence bacterial, fungal, and archaeal communities, although their responses may differ [99,100]. Changes in community composition and predicted microbial functions do not necessarily demonstrate changes in measured nutrient-cycling rates or microbial network stability. Antibiotic residues and trophic interactions represent potential ecological concerns, but evidence from aquatic organisms and rice–fish systems should be treated as contextual rather than direct RCC soil evidence [101,102]. Similarly, nutrient enrichment may influence plant–fungal associations and soil food-web interactions, although the underlying mechanisms require direct verification in RCC soils [103,104]. Future studies should therefore compare microbial groups, diversity indices, management durations, and measured functional processes to clarify the ecological consequences of RCC. Figure 6 further illustrates the potential linkages among RCC management inputs, internal nutrient redistribution, soil ecological processes, and environmental risks, complementing the broader synthesis and future research priorities presented in Figure 5.

## 5. Conclusions

This study combined bibliometric analysis of WoS- and CNKI-indexed publications with a narrative synthesis to examine the research landscape and soil ecological implications of rice–crayfish co-culture (RCC) systems. The bibliometric datasets comprised 108 WoS records and 273 CNKI records retrieved up to September 2025. The keyword co-occurrence networks contained 268 nodes and 657 links for WoS and 247 nodes and 394 links for CNKI, reflecting the range of research topics represented in the two databases. The publication records indicate growing research attention to RCC-related soil issues in recent years, although the observed trends are specific to the indexed literature and should not be interpreted as evidence of comprehensive global coverage or a statistically established transition between research stages.

Based on the reviewed literature, the soil ecological implications of RCC were synthesized across six author-defined themes: soil physical structure and aggregate stability, nutrient cycling and soil fertility, soil organic carbon dynamics and carbon stocks, greenhouse-gas emissions, heavy metals and other pollutants, and microbial communities and functions. Existing studies suggest that crayfish activities, organic matter inputs, and water-management practices may jointly influence soil physicochemical properties and biological processes. Nevertheless, these responses are context-dependent, and potential improvements in soil fertility or carbon accumulation may coexist with environmental concerns related to greenhouse-gas emissions, contaminant mobility, and changes in microbial communities. Therefore, the available evidence does not support a general conclusion that RCC systems are inherently environmentally sustainable under all production conditions.

Several research priorities emerge from this synthesis. Future investigations should strengthen the assessment of coupled nutrient and carbon cycling, integrated risks associated with multiple pollutants, hydrological and nutrient exchanges between field and ditch environments, and differences among integrated rice–aquaculture systems. Greater attention should also be given to long-term soil quality, agricultural productivity, and broader environmental trade-offs under intensive RCC management. Long-term field experiments, coordinated monitoring across regions, and integrated assessments of soil, water, and biological processes are needed to clarify the conditions under which RCC can contribute to sustainable agricultural development. Potential salinity risks associated with irrigation-water quality and drainage conditions also warrant consideration in future assessments, particularly in regions susceptible to soil or groundwater salinization.

## Figures and Tables

**Table 1:** Disciplinary Distribution of Retrieved RCC-Related Literature.

Academic Disciplines and Research Directions	Literature Proportion (%)
Agricultural resource utilization	59.84%
Biological sciences	30.18%
Environmental science and engineering	27.30%
Interdisciplinary	13.91%
Food science and engineering	9.45%
Aquaculture	8.14%
Other topics	6.56%

Note: Percentages were calculated using the combined total of 381 database records (108 WoS and 273 CNKI records) as the denominator, without cross-database deduplication. A single record may be assigned to multiple disciplinary categories; therefore, the sum of the percentages may exceed 100%. These values represent record-based disciplinary distributions rather than proportions of unique publications.

**Table 2:** Selected Publications Related to Rice–Crayfish Co-Culture and Integrated Rice–Aquaculture Systems.

Literature Type	Year	References No.	Title	Journal Source
International Literature	2025	[20]	Rice-crayfish farming increases soil organic carbon sequestration by promoting aggregate protection and microbial necromass accumulation.	Agriculture, Ecosystems & Environment
	2024	[21]	Variations in the profile distribution of soil aggregates and organic carbon under rice-crayfish coculture system in Jianghan Plain, China.	Soil & Tillage Research
	2023	[22]	Agricultural land suitability analysis for an integrated rice-crayfish culture using a fuzzy AHP and GIS in central China.	Ecological Indicators
	2022	[23]	Long-term rice-crayfish-turtle co-culture maintains high crop yields by improving soil health and increasing soil microbial community stability.	Geoderma
	2021	[24]	Life cycle environmental impact assessment of rice-crayfish integrated system: A case study.	Journal of Cleaner Production
	2020	[25]	Soil quality indicators of integrated rice-crayfish farming in the Jianghan Plain, China using a minimum data set.	Soil & Tillage Research
Chinese Literature	2025	[26]	Research of paddy soil dissolved organic matter composition and its binding process with Cd^2+^. (in Chinese)	Acta Pedologica Sinica
	2024	[27]	Research progress and prospect of integrated rice-fish coculture. (in Chinese)	Journal of South China Agricultural University
	2023	[28]	Main Models and Research Progress of Planting-breeding Ecosystem in Rice Field. (in Chinese)	Hybrid Rice
	2022	[29]	Progress in integrated rice-crayfish farming system. (in Chinese)	Journal of Agricultural Science and Technology
	2021	[30]	Effects of the Integrated Rice-crayfish Farming on Nitrogen Accumulation and Quality of Rice. (in Chinese)	Journal of Ecology and Rural Environment
	2020	[31]	Development status and rethinking of the integrated rice-fish system in China. (in Chinese)	China Rice

Note: The publications were selected with reference to citation rankings within each publication year in the WoS and CNKI datasets in September 2025, with one publication presented for each listed year in each literature group. The table provides selected examples of publications related to RCC and integrated rice–aquaculture systems rather than a formal bibliometric citation ranking. No official WoS top-1% highly cited designation or cross-database citation comparison is implied. Broader rice–fish and rice–crayfish–turtle studies are included for contextual comparison.

## Data Availability

The data that support the findings of this study are available from the corresponding author upon reasonable request.
